# Supervisor Narcissism and Time Theft: Investigating the Mediating Roles of Emotional Exhaustion and the Moderating Roles of Attachment Style

**DOI:** 10.3389/fpsyg.2018.02215

**Published:** 2018-11-15

**Authors:** Zhihui Ding, Wenxing Liu, Guanglei Zhang, Huaqiang Wang

**Affiliations:** ^1^School of Public Administration, Zhongnan University of Economics and Law, Wuhan, China; ^2^School of Business Administration, Zhongnan University of Economics and Law, Wuhan, China; ^3^School of Management, Wuhan University of Technology, Wuhan, China; ^4^School of Management, Yangtze University, Jingzhou, China

**Keywords:** supervisor narcissism, emotional exhaustion, attachment avoidance, attachment anxiety, time theft

## Abstract

Although some studies have begun to explore the factors influencing employees’ time theft, it has not been uncommon to link employee time theft to leader personality traits. Based on the conservation of resources theory, this paper examines the influence of supervisor narcissism on employee time theft. It is found that supervisor narcissism positively affects employee time theft via the emotional exhaustion of employees. Further, employee’s attachment styles moderate the mediation effect of emotional exhaustion between supervisor narcissism and employee time theft. This study adds important insights into employee time theft, leader negative traits and the theory and practice of organizational management.

## Introduction

In recent years, deviant behavior at the workplace has been attracting more and more attention from scholars (Berry et al., 2007). Most of the previous studies on this topic had focused on more serious deviation behaviors, e.g., violent and aggressive behaviors (Berry et al., 2007; Cohen, 2016; Jiang et al., 2017). However, past studies have often overlooked hidden, less harmful, but chronic and long-term negative effects, such as time theft, on employee behavior. *Employee* *time theft* is defined as “the propensity of employees to engage in unsanctioned non-work related activities during work time, including off-task activities in the workplace and coming to work late” (Martin et al., 2010). Employee time theft includes any waste of working time such as preoccupation with personal activities on the Internet (e.g., *shopping, watching news, weibo*) and too much chatting with colleagues. Compared with other deviant behaviors, time theft results in no direct harm to other people or organizations; it does not focus on negative motivations (Henle et al., 2010). Yet, time theft is detrimental to organizations. Unfortunately, time theft behavior has not attracted enough attention by scholars. To advance this line of research, we consider it necessary to explore the determinants of employee time theft.

Most of the existing literature discusses the factors influencing time theft from the viewpoints of individual employees (e.g., *personal traits, affect*), the work level (e.g., *work complexity*), and the organizational level (e.g., *organizational commitment*, Henle et al., 2010; Martin et al., 2010; Liu and Berry, 2013; Brock Baskin et al., 2017). Until recently, some scholars have tried to explore the mechanism of time theft from the perspective of leadership style, e.g., empowering leadership styles (Lorinkova and Perry, 2017). However, as far as we know, no study has empirically explored the relationship between dark traits of leadership (e.g., *narcissism*) and employee time theft. The negative personality trait of narcissism has often been associated with aspects of leadership such as leader emergence (Grijalva et al., 2015) and diversities of leadership behavior (Rosenthal and Pittinsky, 2006). Narcissistic leaders usually strive for personal success, power and take delight in self-centered perspectives (Rosenthal and Pittinsky, 2006). Therefore, we believed that supervisor narcissism is perceived as supervisors with narcissistic personality traits, and could be leading in an autocratic, inconsiderate, exploitative and self-serving manner, thereby displaying unethical, despotic leadership (Grijalva et al., 2015). Several studies have shown that narcissism tends to motivate workplace deviant behavior (Ouimet, 2010). O’Boyle et al. (2012) also confirmed that narcissism is related to deviant behavior, immorality and exploitation at the workplace, e.g., cheating, lack of honesty and even criminal behavior. Thus, it is of high value to investigate the influence of supervisor narcissism on employees’ time theft.

In order to better understand the relationship between supervisor narcissism and time theft, this paper seeks to explain it by applying conservation of resources theory (COR, Hobfoll, 1989). Specifically, it is proposed that supervisor narcissism more often leads to employee time theft owing to the latter’s emotional exhaustion. *Employee emotional exhaustion* is defined as an individual’s state of fatigue after overuse of mental and emotional resources, which is a result of the individual’s stress response to a stressor in the workplace. COR theory posits that employees facing negative emotions consume their individual resources (Hobfoll, 1989) and that emotional exhaustion is most likely to occur in this situation. In order to avoid excessive loss of resources, individuals do take some actions in response to save individual resources, time theft is considered a good way (Ketchen et al., 2008). Furthermore, different individuals will have different reactions in the face of negative emotions or pressure. Individual characteristics often influence individual pressure perception (Cropanzano et al., 2003), such as attachment style. *Employee attachment style* is defined as an individual’s natural tendency to seek others when in need, while the individual develops different strategies for seeking proximity based on earlier experiences (Bowlby, 1973). The tendency is generally divided into two basic dimensions: attachment avoidance and attachment anxiety (Brennan et al., 1998). Many studies have noted that employee attachment style can influence work mood, job satisfaction, trust, job burnout, and mental health in the workplace (Sumer and Knight, 2001; Little et al., 2011). Keeping these observations in mind, we further propose that the impact of supervisor narcissism on time theft via emotional exhaustion can be moderated by employee attachment style.

**FIGURE 1:**
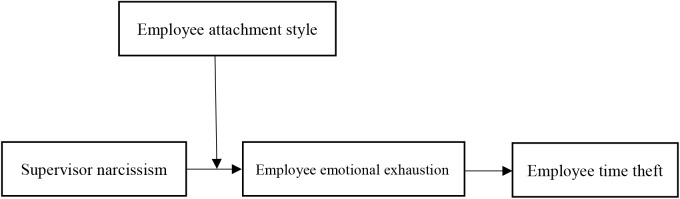
Theoretical model.

In sum, our research makes three contributions to literature on narcissism, leadership and time theft. First, it explores the influence of supervisor narcissism on employee time theft and further delves into studying the relationship between personality traits and employee time theft. Second, it clarifies how supervisor narcissism affects employee time theft via emotional exhaustion. Third, attachment style as an important part of individual characteristics, have an important influence on stress perception. This paper examines the moderating effect of individual attachment styles on the relationship between supervisor narcissism and time theft. The overall theoretical model is presented in Figure 1.

## Theory and Hypotheses

### Supervisor Narcissism and Employee Time Theft

Following the introduction of the notion of narcissism into the field of organizational behavior, some studies have found that many leader behaviors are actually influenced by the narcissistic personality traits of the leaders (de Vries and Miller, 1985). Ouimet (2010) pointed out that narcissistic leaders exhibit charisma, excessive pursuit of self-influence, false motives and, in the process, suppress employee performance. Some studies further confirmed that narcissistic leaders are characterized by ego, arrogance, aggression and possessiveness, and that these behaviors are often aggressive and destructive (Campbell et al., 2011). Researchers have shown that narcissistic leaders often ignore, or even dislike, an employee whose views are inconsistent with theirs. Indeed, they are likely to threaten or suppress employees who offer opinions that differ from theirs (Grijalva et al., 2015). Such leaders are willing to exploit subordinates and attribute subordinates’ successes to themselves while blaming their failures and shortcomings on their employees (Locke, 2009). Some scholars have pointed out that the negative influence of leadership narcissism is far greater than its positive effect (Grijalva et al., 2015). The negative impact of narcissism on employees has been supported by a large number of empirical studies by invoking a lack of trust and sincerity, creating a destructive working environment, reducing employee job satisfaction, and triggering a counterproductive workplace behavior (Ouimet, 2010; Hochwarter and Thompson, 2012; Cohen, 2016).

According to COR theory, when individuals perceive some stressors, they will try to protect, maintain and obtain the necessary resources to help them achieve their own goals (Hobfoll, 2001). This process is guided by two principles. First, the loss of resources is more important than the entailing resource acquisition, so they may choose to give up the opportunity to gain other resources in order to avoid losing previous resources. Second, they choose to invest on new resources to avoid the continuous loss of resources (van Woerkom et al., 2016). As a kind of passive aggressive behavior of the organization, employee time theft is very suitable for those who are disillusioned, frustrated and not valued by the organization. Such behavior is not only concealed from the organization but also is less dangerous and risky than other deviant behaviors (Ketchen et al., 2008). By doing so, employees can release their pressure arising from self-defeat and helplessness, thus preserving their valuable resources without being randomly consumed by the organization (Mitchell and Ambrose, 2007). COR theory suggests that employees often feel frustrated and helpless when they encounter unfriendly behavior from the leader (such as criticize, attack), they start perceiving the loss of their resources (e.g., *psychological resources*). Individuals may then choose to give up other resources (e.g., *interpersonal relationship with supervisor*) in order to avoid losing some resources (e.g., *psychological resources*; Aryee et al., 2008). Building on previous findings on workplace deviant behavior, leadership behavior is considered to be an important situational factor inducing workplace deviant behavior (Sharma, 2017). In the case of supervisor narcissism, due to the unequal power and status between the supervisor and the subordinate, it is generally unlikely that the subordinate chooses an open way to oppose their direct leader, but instead, adopt a more secure and invisible way to vent his/her frustration (Liu and Berry, 2013), e.g., engage in time theft. Thus, time theft is also a kind of feedback behavior in which employees are disappointed, frustrated, and perceived by the organization to despise themselves. It is also an act of passive retaliatory organization or leadership. Taking these considerations together, we proposed the following hypothesis:

***Hypothesis 1: Supervisor narcissism is positively related with employee time theft.***

### The Mediating Role of Employee Emotional Exhaustion

Prior research findings have shown that emotional exhaustion is usually a result of the individual’s stress reaction to stressors at the workplace (Maslach et al., 2001). Many scholars have found that emotional exhaustion not only leads to a loss of self-esteem, frustration, nervousness and irritability on the part of the employee, but also leads to reduced job involvement and reduced performance (Aryee et al., 2008). Emotional exhaustion is a state of exhaustion of emotional and physiological resources, a feeling of personal emotional resources and the exhaustion of physiological resources associated with them (Lam et al., 2017). Studies have shown that when employees experience unfriendly behavior, they may exhibit a behavior that harms the interests of the organization, so time theft becomes a logical way for employees to respond to pressures from the organization (Henle et al., 2010). Time theft encourages employees to temporarily escape from their negative emotions or circumstances (Martin et al., 2010).

According to COR theory, the resources of an individual are limited (e.g., *psychological resources*). On the one hand, it may lead to the depletion of one’s own resources if he/she invests them continuously for work. On the other hand, individual intrinsic motivation provides the protection necessary to expand one’s own resources. However, the individual’s resources are limited. The contradiction between these two considerations encourage the individual to choose to reduce or stop resource inputs for protecting the depletion of their own resources (van Woerkom et al., 2016). Leaders with narcissistic personality traits often blame, threaten, or even demoralize their employees while interacting with them, thus causing employees to develop negative emotions (Grijalva et al., 2015). Klotz and Neubaum (2016) have also pointed out that narcissistic leaders not only abuse their power in the organization to reinforce their personal needs, but also bully and oppress their employees. The impolite behavior of the leader toward the employee negatively affects the employee’s psychology, causing greater psychological pressure. The result is, this creates negative emotions of resistance in the employees’ mind, which further leads to emotional exhaustion of the employee, or even self-denial (Hobfoll, 2001). Therefore, in order to avoid further loss of their own resources, employees may choose some kind of time theft behavior in response to the narcissistic behavior against the narcissistic leader. We proposed the following hypothesis:

***Hypothesis 2: Supervisor narcissism is positively related with employee emotional exhaustion.***
***Hypothesis 3: Employee emotional exhaustion mediates the relationship between supervisor narcissism and employee time theft.***

### The Moderating Role of Employee Attachment Style

Harms et al. (2017) found that an individual’s characteristics present an important influence factor that affects individual pressure perception, especially individual personality characteristics. Different individual traits have a different understanding of the same stressors, e.g., highly neurotic employees who are more sensitive to stress (Bakker et al., 2006). Also, an employees’ agreeableness traits are positively correlated with job stress (Byrne et al., 2015). Some studies have found that attachment is a relatively stable personality trait, and individual attachment types also affect individual aspects such as interpersonal relationship, emotion and marriage (Bowlby, 1973). Therefore, the employee’s attachment style is an important trait variable influencing his/her time theft behavior. Brennan et al. (1998) divide adult attachment into two dimensions. The first is attachment avoidance, which refers to the individual not only showing fear of interpersonal intimacy, but also that the intimacy or dependence that others show on them in interpersonal interaction makes them feel uncomfortable, and even shows rejection. The second is attachment anxiety, which refers to the individual showing fear of being rejected and abandoned by others in interpersonal interactions charged with tension, anxiety, etc. Therefore, we argue that employee attachment style will moderate the relationship between supervisor narcissism and employee emotional exhaustion.

Research has highlighted the fact that employees’ attachment styles significantly affect workplace emotion, job satisfaction, trust, job burnout, and mental health at the workplace (Sumer and Knight, 2001; Little et al., 2011). Employees with high levels of attachment avoidance tend to be self-reliant, thus generally keeping the leader at arm’s length and not overly emphasizing a dependency relationship with the leader (Frazier et al., 2015). While employees with high levels of attachment anxiety exhibit the need for close interpersonal interaction with the leader, this need takes on a “double face.” On the one hand, employees are benefited by the close interpersonal relationship with the leader. On the other hand, because the leadership is unable to satisfy or hurt the staff, they will feel more anxious and more negative (Richards and Schat, 2011). COR theory holds that the resources of individuals are limited, and the interpersonal interaction consumes a lot of resources such as time, psychology and physiology. With the loss of individual resources, individuals acquire new resources as much as possible to preserve or maintain balance in resources. Specifically, the general individual has two tendencies, resource conservation tendencies and resource acquisition tendencies, so individuals in the face of pressure scenarios are more willing to choose the strategy of resource conservation (Cohen, 2016).

Therefore, when employees are confronted with unfriendly behaviors such as humiliation, abuse and debasement by leaders with narcissistic personality, the individuals with attachment avoidance become skeptical about the motivations and intentions of the leaders themselves, distrust the leader, and become unwilling to have too much interpersonal interaction with the leader. By doing so, they perceive less pressure, anxiety or resentment. Although their own resources are also lost a little, the level of emotional exhaustion of employees will be lower. Because individuals with attachment avoidance experience no excessive loss of their own resources, the negative influence of supervisor narcissism does not amplify the individual’s emotional exhaustion, and they tend not to engage in time theft behavior. On the contrary, individuals with attachment anxiety interact frequently with leadership to satisfy their own needs, so they become overly sensitive to the feedback of leadership behavior (Maslyn et al., 2017). As a result, when leadership is unable to return their support adequately, the stress and anxiety they perceive will be greater. This leads to greater consumption of their resources, and may even cause psychological and behavioral harm, resulting in emotional exhaustion of the individual. Because of the greater loss of resources of individuals arising from attachment anxiety, the negative effects of leadership narcissism further amplify the adverse effects of emotional exhaustion. This leads to the individuals engaging in time theft in order to save or restore their own resources. Based on the above analysis, we propose hypothesis 4 and hypothesis 5.

***Hypothesis 4a: Employee attachment avoidance will moderate the relationship between supervisor narcissism and employee emotional exhaustion. Specifically, the positive relationship between supervisor narcissism and employee emotional exhaustion will be weaker for respondents who report higher levels of employee attachment avoidance.***
***Hypothesis 4b: Employee attachment anxiety will moderate the relationship between supervisor narcissism and employee emotional exhaustion. Specifically, the positive relationship between supervisor narcissism and employee emotional exhaustion will be stronger for respondents who report higher levels of employee attachment anxiety.***

Hypothesis 1, 2, 3, and 4 can be combined to produce hypothesis 5.

***Hypothesis 5a: Employee attachment avoidance moderates the mediation effect of employee emotional exhaustion pertaining to the relationship between supervisor narcissism and employee time theft, such that the mediation effect is lower when employee attachment avoidance is high.***
***Hypothesis 5b: Employee attachment anxiety moderates the mediation effect of employee emotional exhaustion pertaining to the relationship between supervisor narcissism and employee time theft, such that the mediation effect is higher when employee attachment anxiety is high.***

## Materials and Methods

### Sample and Procedure

The survey samples came mainly from Shenzhen, Shanghai, Wuhan, and Jingzhou in China. We first contacted the head of the business, and asked for his permission. We told the head of the business that the questionnaires were confidential and data used only for scientific research. Then, we sent the questionnaires directly and did not allow the company managers to participate in the management of the survey. The questionnaires were accompanied by a letter describing the study, explaining that participation was completely voluntary and guaranteeing the confidentiality of the response. At the same time, in order to increase the level of participation, we asked participant to complete the questionnaire within normal working hours and to return the surveys directly to our study team. All respondents signed an informed consent and agreed to participate in the study. There was no unethical behavior during the research process because this study did not involve human clinical trials or animal experiments. Therefore, we were relieved of further ethics committee approval. Ethical approval was not required for this study in accordance with the recommendations of Zhongnan University of Economics and Law’s Human Research Ethics Committee.

In order to reduce the impact of sample homology bias, we collected data from the study in two stages. Survey 1, was designed to record employees’ gender, age, education, tenure, along with their perceptions of their direct supervisor’s narcissism, their own emotional exhaustion and attachment style. Survey 2 was designed to measure employees’ time theft. Survey 1 was completed by the subordinates on August 1, 2017 and Survey 2 was completed 1 month later, we chose to use a short period of time for this result because we felt that it would be less likely for employees to recall the amount of time theft they had committed during an extensive historical period (Pearson et al., 1991). For data collection, each survey form was numbered. Once training of the investigators was completed with the assistance of the enterprise leader, questionnaires were issued. We sent out 300 questionnaires, recovery of valid questionnaires 214, recovery 71.3%. In the survey sample, respondents under 25 years of age accounted for 25.24%, respondents aged 25–29 accounted for 59.81%, respondents aged 30–34 accounted for 12.62%, respondents aged 35–39 accounted for 1.4%, respondents aged 40 or above accounted for 0.93%. The age of respondents was 28.56 years (*SD* = 0.72) and 35.05% were women. Education level in high school and below the respondents accounted for 5.14%, college accounted for 8.41%, undergraduate accounted for 77.57%, Master’s degree and above accounted for 8.88%. Respondents with <1 year tenure accounted for 31.30%, with 1–3 years accounted for 26.64%, with 3–5 years accounted for 24.77%, with 6–10 years accounted for 14.95%, and over 10 years accounted for 2.34%.

### Measurement

#### Supervisor Narcissism

Supervisor narcissism was measured using the scale developed by Hochwarter and Thompson (2012), which is based on certain personality traits identified, following leadership research. There are six measurement items, each rated on a 5-point scale ranging from 1 (strongly disagree) to 5 (strongly agree) such as “My leader is a very self-centered person.” In this study, the Cronbach’s alpha was set at 0.90.

#### Employee Emotional Exhaustion

We measured emotional exhaustion using the scale developed by Maslach and Jackson (1981). There were four measurement items, which were rated on a 5-point scale ranging from 1 (strongly disagree) to 5 (strongly agree) such as “I feel exhausted when I’m off duty.” In this study, the Cronbach’s alpha was set at 0.76.

#### Employee Attachment Style

We used the scale developed by Zhao and Sun (2014) to measure attachment style. The scale contains two dimensions, namely attachment avoidance and attachment anxiety, each with six items. The items were rated on a 5-point scale ranging from 1 (strongly disagree) to 5 (strongly agree), such as attachment avoidance: “I find myself holding back when friends start to want to be close to me,” attachment anxiety: “I feel a bit anxious and upset if I don’t have friends.” In this study, the Cronbach’s alpha was set at 0.86 (attachment avoidance) and 0.83 (attachment anxiety).

#### Employee Time Theft

We measured time theft using the scale developed by Bennett and Robinson (2000). There were three measurement items, which were rated on a 5-point scale ranging from 1 (never) to 5 (very often) such as “Dealing with personal things at work rather than working for the boss.” In this study, the Cronbach’s alpha was set at 0.78.

### Control Variables

Some scholars have confirmed that the gender (1 = female, 2 = male), age (in years), education level and tenure (in years) have an influence on the unethical behavior of employees (Whitley et al., 1999; Oreg and Berson, 2011). Therefore, these variables were used as controlling variables.

## Data Analysis and Results

To verify the validity of the hypothesis made in this study, we used SPSS 23 and AMOS 22.0 to analyze the data.

### Confirmatory Factor Analysis

This study used Amos 22.0 to carry out confirmatory factor analysis to validate each variable. We selected four indices: χ^2^/d*f*, root mean square error of approximation (RMSEA), comparative fit index (CFI), and Tucker–Lewis index (TLI, see Table 1). The table 1 shows that the five-factor model yielded the best fit (χ^2^/d*f* = 1.94, RMSEA = 0.07, CFI = 0.90, TLI = 0.89). Alternatives four-, three-, two-, and one-factor of the nested model were significantly worse than the five-factor nested model. Comprehensive Table 1 shows that the five variables have good discriminant validity, and therefore the analyses of the relationship between them can be further expanded.

**Table 1:** Confirmatory factor analyses.

Models	χ^2^	d*f*	χ^2^/d*f*	RMSEA	CFI	TLI	Δχ^2^	Δd*f*	*P*
Five-factor Model (SN,EE,AV,AN,TT)	512.90	265	1.94	0.07	0.90	0.89			
Four-factor Model (SN,EE,AV+AN,TT)	1024.92	269	3.81	0.12	0.71	0.67	512.02	4	<0.001
Three-factor Model (SN,EE+AV+AN,TT)	1145.04	272	4.21	0.12	0.66	0.63	632.14	7	<0.001
Two-factor Model (SN,EE+AV+AN+TT)	1198.79	274	4.38	0.13	0.64	0.61	685.89	9	<0.001
One-factor Model (SN+EE+AV+AN+TT)	1597.62	275	5.81	0.15	0.49	0.44	1084.72	10	<0.001

*SN, supervisor narcissism; EE, emotional exhaustion; AV, attachment avoidance; AN, attachment anxiety; TT, time theft; RMSEA, root mean square error of approximation; CFI, comparative fit index; TLI, Tucker–Lewis index*.

### Correlation Analysis of Variables

The means, standard deviations, and correlations coefficients of the study variables are shown in Table 2. The table shows that, supervisor narcissism has significantly correlated with both employee emotional exhaustion (*r* = 0.34, *p* < 0.01) and employee time theft (*r* = 0.43, *p* < 0.01). Furthermore, employee emotional exhaustion is significantly and positively related to employee time theft (*r* = 0.53, *p* < 0.01).

**Table 2:** Means, standard deviations, and intercorrelations of variables.

Variable	*M*	*SD*	1	2	3	4	5	6	7	8
1. Gender	1.65	0.48								
2. Age	1.93	0.72	-0.20^∗∗^							
3. Education	2.90	0.61	-0.04	-0.08						
4. Tenure	2.30	0.68	-0.07	0.67^∗∗^	-0.29^∗∗^					
5. Supervisor narcissism	2.69	0.78	-0.03	-0.08	-0.20^∗∗^	-0.09				
6. Emotional exhaustion	2.82	0.77	-0.16^∗^	-0.05	-0.01	0.03	0.34^∗∗^			
7. Attachment avoidance	2.69	0.86	-0.03	-0.06	-0.07	-0.10	0.33^∗∗^	-0.02		
8. Attachment anxiety	2.52	0.79	-0.14^∗^	0.01	0.04	0.01	0.43^∗∗^	0.54^∗∗^	0.19^∗∗^	
9. Time theft	2.58	0.88	-0.26^∗^	-0.10	-0.03	-0.08	0.43^∗∗^	0.53^∗∗^	-0.03	0.58^∗∗^

^∗^*p < 0.05, ^∗∗^*p* < 0.01*.

### Results From Hypothesis Tests

Based on the methods recommended by Baron and Kenny (1986), we examined whether employee emotional exhaustion mediates the relationship between supervisor narcissism and employee time theft. The results are presented in Table 3. Note that the results for Model 2 show that supervisor narcissism has significantly positive influence on employee time theft, thus Hypotheses 1 was supported. Likewise, the results for Model 3 show that supervisor narcissism has a significantly positive influence on employee emotional exhaustion, so we can conclude that Hypotheses 2 has also been supported. Finally, as for Model 4, we added both supervisor narcissism and employee emotional exhaustion into the regression equation. The regression results showed that compared with Model 2, the influence of supervisor narcissism on employee time theft was weaker, but both supervisor narcissism and employee emotional exhaustion still had a significant influence on employee time theft. Therefore, it can be concluded that employee emotional exhaustion mediates the relationship between supervisor narcissism and employee time theft. This supports hypothesis 3.

**Table 3:** Results of regression analysis.

Variable	Time theft	Emotional exhaustion	Time theft
	
	Model 1	Model 2	Model 3	Model 4
Gender	-0.29**	-0.27**	-0.17**	-0.20**
Age	-0.15	-0.15	-0.17*	-0.08
Education	-0.06	0.05	0.10	0.01
Tenure	-0.02	0.05	0.19*	-0.03
Supervisor narcissism		0.43**	0.36**	0.29**
Emotional exhaustion				0.39**
*R*^2^	0.09	0.26	0.16	0.39
Δ*R*^2^		0.17		0.13
*F*	5.314**	14.79**	8.15**	22.21**

^∗^*p < 0.05, ^∗∗^*p* < 0.01, ^∗∗∗^*p* < 0.001*.

We used hierarchical regression methods to test hypothesis 4a and 4b. The regression results are shown in Table 4. Note that Models 1, 2, and 3 are concerned with the moderating effects of employee attachment avoidance style while Models 1, 4, and 5 are concerned with those of employee attachment anxiety style. In order to eliminate the effect of collinearity, based on the methods recommended by West et al. (1996), supervisor narcissism and employee attachment style were standardized, respectively, when constructing the product of the moderate variables. As shown in Table 4, Model 3 shows a significant interaction coefficient (β = -0.13, *p* < 0.05) between supervisor narcissism and employee attachment avoidance, which explains the 2% variance. Our results have shown that attachment avoidance plays a negative role in determining supervisor narcissism and employee emotional exhaustion. This meant that Hypotheses 4a was supported. Model 5 showed a significant interaction coefficient (β = 0.22, *p* < 0.001) between supervisor narcissism and employee attachment anxiety; this significantly explains the 4% variance. Our results also showed that employee attachment anxiety plays a positive role in the relationship between supervisor narcissism and employee emotional exhaustion. Thus, Hypotheses 4b was supported.

**Table 4:** Results of regression analysis.

Variable	Emotional exhaustion				
	Model 1	Model 2	Model 3	Model 4	Model 5
Gender	-0.19**	-0.17**	-0.17**	-0.11	-0.09
Age	-0.18	-0.17	-0.17*	-0.15	-0.17*
Education	0.02	0.10	0.07	0.03	0.03
Tenure	0.14	0.18*	0.18*	0.14	0.13
Supervisor narcissism		0.41***	0.40***	0.15*	0.17**
Attachment avoidance		-0.14*	-0.12		
Attachment anxiety				0.46***	0.43***
Supervisor narcissism^∗^ Attachment avoidance			-0.13*		
Supervisor narcissism^∗^ Attachment anxiety					0.22***
*R*^2^	0.04	0.18	0.20	0.33	0.37
Δ*R*^2^		0.14	0.02	0.29	0.04
*F*	2.33	7.64***	7.25***	16.86***	17.60***

^∗^*p < 0.05, ^∗∗^*p* < 0.01, ^∗∗∗^*p* < 0.001*.

In addition, our research followed Preacher et al. (2007) recommended method of testing for moderated mediation and used the Bootstrap method to test the model by the statistical software SPSS PROCESS 2.16. After controlling for the variables of employee gender, employee age, employee education level and employee tenure, the results showed that the path coefficient between supervisor narcissism and employee emotional exhaustion was 0.28 (*p* < 0.001), and the path coefficient between employee emotional exhaustion and employee time theft was 0.45 (*p* < 0.001). The indirect effects of supervisor narcissism on employee time theft via employee emotional exhaustion was significant (β = 0.124, *p* < 0.001), and bootstrap set to 5,000 times, 95% confidence interval (CI) [CI = 0.084, 0.180] did not include zero. Thus, hypothesis 3 was supported.

Furthermore, the results showed that the interaction between supervisor narcissism and employee attachment avoidance was significantly related to employee emotional exhaustion (β = -0.095, *p* < 0.05). Thus, hypothesis 4a was supported. Moving on to testing conditional indirect effect, when the attachment avoidance takes two different conditional values, that is, the mean value plus a standard deviation and minus a standard deviation, the difference in indirect effect of employee emotional exhaustion between supervisor narcissism and employee time theft was significant. Specifically, under the condition of high-level employee attachment avoidance (1 SD above the mean), the indirect effect of employee emotional exhaustion was significant (β = 0.089, *p* < 0.01), and when the bootstrap was set to 5,000 times, the 95% confidence interval was [0.047, 0.147], not including zero. And the indirect effect of employee emotional exhaustion was significant for the condition of low-level employee attachment avoidance (1 SD below the mean) (β = 0.156, *p* < 0.001), bootstrap set to 5,000 times, the 95% confidence interval was [0.107, 0.223], again not including zero. The difference in mediating effect between two conditions was significant (β = -0.066, *p* < 0.05), and the confidence interval of 95% was [-0.129, -0.022] with zero not being included. Thus, hypothesis 5a was supported.

The results also showed that the interaction between supervisor narcissism and employee attachment anxiety was significantly related to employee emotional exhaustion (β = 0.159, *p* < 0.001). Thus, hypothesis 4b was supported. When attachment anxiety takes two different conditional values, that is, the mean value plus a standard deviation and minus a standard deviation, the difference in the indirect effect of employee emotional exhaustion between supervisor narcissism and employee time theft was significant. Under high levels of employee attachment anxiety (1 SD above the mean), the indirect effect of employee emotional exhaustion was significant (β = 0.061, *p* < 0.05), and when the bootstrap was set at 5,000 times, the 95% confidence interval was [0.028, 0.112], not including zero. The indirect effect of employee emotional exhaustion was not significant for the condition of low-level attachment anxiety (1 SD below the mean) (β = 0.002, *p* < 0.001), bootstrap set to 5,000 times, the 95% confidence interval was [-0.019, 0.025], including zero. The difference in the mediating effect between the two conditions was significant (β = 0.059, *p* < 0.01), and the 95% confidence interval was [0.029, 0.104] with zero not being included. Thus, hypothesis 5b was supported.

**FIGURE 2:**
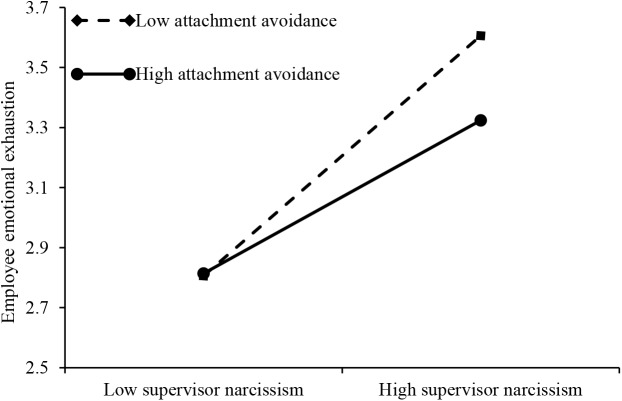
Moderating effect of employee attachment avoidance on relationship between supervisor narcissism and employee emotional exhaustion.

**FIGURE 3:**
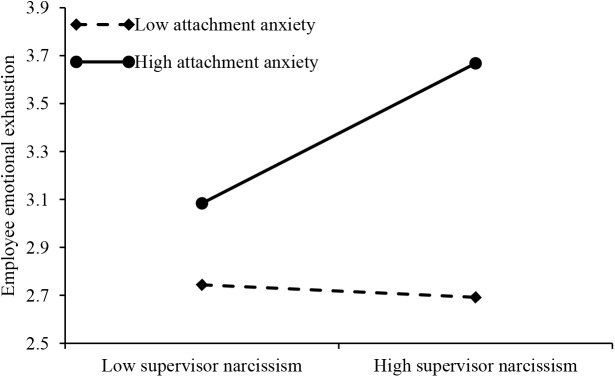
Moderating effect of employee attachment anxiety on relationship between supervisor narcissism and employee emotional exhaustion.

By drawing the moderated effect graph of the attachment style, it is possible to clearly identify the moderate effects (see Figures 2, 3). Figure 2 shows that the sample was divided into high and low groups according to the mean values of employee attachment avoidance. The regression equations were calculated separately. Compared with the condition of low-level employee attachment avoidance, the mediating role of employee emotional exhaustion in supervisor narcissism and employee time theft could be attenuated under conditions of high-level employee attachment avoidance. Figure 3 shows that the sample was divided into high and low groups according to the mean value of employee attachment anxiety. The regression equations were calculated separately. Compared with the condition of low-level employee attachment anxiety, the mediating role of employee emotional exhaustion in supervisor narcissism and employee time theft could be strengthened under conditions of high level of employee attachment anxiety.

## Discussion

Based on COR theory, our study research has thrown light on how supervisor narcissism affects employee time theft behavior via employee emotional exhaustion. Our results have shown that supervisor narcissism leads to employees’ emotional exhaustion and promotes their time theft. In addition, we have found that employees’ gender negatively related with employee time theft, employees’ attachment style moderates the mediation effect of employee emotional exhaustion between supervisor narcissism and time theft. Specifically, employees’ attachment avoidance style negatively moderates the mediation effect of employee emotional exhaustion, while employee attachment anxiety style positively moderates the mediation effect of employee emotional exhaustion.

### Theoretical Implications

First, our study has validated the positive influence of supervisor narcissism on employee time theft and provided a new way of explaining time theft. While discussing the influencing factors of employees’ time theft, most previous studies had adopted the perspective of employee, e.g., employee attitude and organizational identity (Ketchen et al., 2008; Martin et al., 2010). Most of them had ignored other important variables such as organizational situation of leadership, especially the dark personality traits of leadership. Likewise, previous studies on supervisor narcissism had found that the diffusion effect of the negative influence of supervisor narcissism could trigger the negative behavior of employees (Campbell et al., 2011). Our research has empirically examined the influence of supervisor narcissism on employees’ time theft. It has not only provided a novel way of explaining employees’ time theft behavior but also enriched theoretical research on the negative influence of supervisor narcissism.

Second, by introducing employee emotional exhaustion as a mediating variable, we have found the conduction mechanism of supervisor narcissism and employees’ time theft. We have analyzed the mechanism of the influence of supervisor narcissism on employees’ time theft through employee’s emotional exhaustion from the perspective of the COR. We have also shown that, the negative behavior presented by leaders with narcissistic personality traits on employee behavior is not one stroke, but a process. When employees face the negative behavior of supervisor narcissism, negative emotional experiences consume their psychological resources, which in turn lead to emotional exhaustion or collapse on the part of the employee (Mackey et al., 2017). After employees experience emotional exhaustion, these negative emotions not only erode their commitment to work, but also lead to employee perfunctory performance (Ketchen et al., 2008). This results in time theft behavior. Thus, our study has also contributed to emotional exhaustion literature by enriching COR theory and opening up the otherwise “black box” relationship between supervisor narcissism and employee time theft.

Third, this study has examined the moderating effect of employee attachment style and explored the differences between individual attachment styles in the process of their time theft behavior. It has shown that attachment avoidance style negatively moderates the mediation role of employee emotional exhaustion between supervisor narcissism and employee time theft, while attachment anxiety style positively moderates the mediation role of employee emotional exhaustion between supervisor narcissism and employee time theft. The conclusion of this study on attachment style helps determine the boundary condition of the influence of supervisor narcissism on employees’ time theft. On the other hand, by considering differences in the influence of individual characteristics on employees’ emotional exhaustion, it has provided a new perspective and new thinking for the research field of employees’ time theft.

### Limitations and Future Research

Due to certain issues related to our research conditions, our research has some limitations. First of all, our data were derived from Chinese enterprises. When taking into account the cross-cultural differences, whether the conclusions reached in this study are applicable to Western countries needs investigation. Secondly, based on the perspective of COR, our study has analyzed the mediating roles of supervisor narcissism and employee time theft by introducing employee’s emotional exhaustion and selecting employee attachment style as the boundary of the research model. Future researchers may try to explore other factors that affect employee time theft, such as leadership style or psychological empowerment, and further deepen the theoretical understanding of employee time theft. Thirdly, we used employee gender (1 = female, 2 = male) as a control variable to examine its influence on employee time theft. The results showed that employees’ gender negatively related with employee time theft. However, previous research suggests that men are more likely than women to engage in minor criminal behavior, excessive alcohol consumption, gambling, and unprovoked aggression (Whitley et al., 1999; Jaffee and Hyde, 2000). We recommend future research to examine the role of gender differences in different types of deviant workplace behavior. Finally, this paper has focused only on the negative aspects of employee time theft from the organization’s point of view. However, previous studies have proposed that employees’ time theft also has some benefits for the organization (Brock Baskin et al., 2017). For example, chats between colleagues can promote team cohesion. In the future, we will do well to further verify under what circumstances the time theft of employees will promote team building and organizational development.

### Managerial Implications

First, it has been helpful to understand the recessive harm of supervisor narcissism. The negative effects of supervisor narcissism have been confirmed by many scholars, but most of them dwelled on overt hazards (Campbell et al., 2011; Grijalva et al., 2015). Our study has found that employees’ time theft is a common but potentially costly deviant behavior. Which concealment is strong, not easily detected by the organization’s managers. Once the organization forms a lazy atmosphere, it may lead to organizational failure. Therefore, leaders should not only realize the hidden dangers of narcissism, but also strive to improve the organization’s sense of crisis.

Second, it is helpful to interfere with the emergence of employees’ time theft. Our research has found that when employees face negative emotions, they lose bits or some of their individual resources, and then choose time theft behavior to protect their own resources. Therefore, the key to reducing employee time theft is to reduce the loss of staff resources, and to prevent organizations’ negative impact on employees, by either infiltration or diffusion. More concretely, the organization should strengthen the behavior of the narcissistic leader control, reduce the impact of their behavior intensity. On the other hand, the organization should control employees’ emotional exhaustion, timely psychological guidance to the staff of the large consumption of mental resources, and actively give resources to supplement, and then avoid the expansion of negative emotion.

Finally, it is good for the organization to fine-tune management staff. Studies have shown that individuals with different attachment styles react differently when faced with negative behavior from their leader. Therefore, in the process of organizing employee management, it is necessary to focus on monitoring employees with attachment anxiety. One way is to encourage them to participate in internal cooperation and exchanges. Additionally, managers must also strive to create a good working atmosphere, reduce the emotional exhaustion of individuals with attachment anxiety, and improve organizational efficiency.

## Conclusion

In this study, we have focused mainly on the relationship between supervisor narcissism and time theft. Our results have refined our understanding of how supervisor narcissism affects time theft. More concretely, supervisor narcissism leads to employees’ emotional exhaustion and promotes time theft on their part. Employee’s attachment style moderates the mediation effect of emotional exhaustion between supervisor narcissism and employee time theft.

## Author Contributions

ZD, WL, and GZ designed and adopted the study, wrote the paper. HW wrote the paper. All the authors discussed and commented on the final version of the manuscript. All the authors critically reviewed content and approved final version for publication.

## Conflict of Interest Statement

The authors declare that the research was conducted in the absence of any commercial or financial relationships that could be construed as a potential conflict of interest.
